# A review of the *sarawakensis* species group of the ground beetle genus *Orthogonius* MacLeay, 1825 (Coleoptera, Carabidae, Orthogoniini)

**DOI:** 10.3897/zookeys.594.8768

**Published:** 2016-05-30

**Authors:** Mingyi Tian, Thierry Deuve

**Affiliations:** 1 Department of Entomology, College of Agriculture, South China Agricultural University, Wushan, Guangzhou, 510642, China; 2 Muséum national d’Histoire naturelle, Département de Systématique & Évolution, Entomologie, C.P. 50, 57 rue Cuvier, F–5231 Paris cedex 05, France

**Keywords:** Ground beetle, termitophilous, taxonomy, new species, Southeast Asia

## Abstract

The *sarawakensis* species group of the termitophilous carabid genus *Orthogonius* MacLeay, 1825 is defined and reviewed. Members of this species group are distributed in Southeast Asia and represented by four species, including two new species: *Orthogonius sabahicus* **sp. n.** (Sabah, northern Borneo, Malaysia) and *Orthogonius morvanianus* **sp. n.** (southern Thailand). A key to all species of the species group is also provided.

## Introduction

As part of the series works on the dominant genus *Orthogonius* MacLeay, 1825 in the tribe Orthogoniini, the *lancangjiang* and *baconii* species groups have been reviewed respectively (Tian and Deuve 2013, 2016). In this paper, the *sarawakensis* species group is dealt with.

The members of the *sarawakensis* species group are medium to large sized, broad and brown ground beetles having large and prominent eyes, rounded off pronotal hind angles, flat intervals, and median and posterior setiferous pores in the 3^rd^ elytral interval located on median portion of the interval, instead of close to the 2^nd^ stria as in other *Orthogonius* species.

All of the four species of this group are distributed in Southeast Asia. The first species of this group is *Orthogonius sarawakensis* Tian & Deuve, 2006 recorded from Sarawak, northern Borneo, eastern Malaysia. Then, the second species *Orthogonius perakicus* Tian & Deuve, 2007 was described from Perak, western Malaysia. In the present paper, further two new species are discovered from Sabah, northern Borneo, Malaysia and from Khao Sok NP, southern Thailand respectively, and hereinafter described.

## Material and methods

All specimens for this study are dry and mounted material. Dissection and observation of the specimens were made using a WILD M32 binocular microscope. Detailed descriptions are provided for the new species, while only diagnostic character states are given for the known species. Habitus and male genital illustrations for all species are also presented. Digital photographs were taken and processed as in Tian and Deuve (2013).

Body length was measured from apex of right mandibles to apex of elytra; body width = width of elytra.

Abbreviations of measurements used in the text are as followings:

HL
 head length (from apex of right mandible to base of vertex) 


HW
 head width (maximum distance across head, including eyes) 


PL
 length of pronotum (measured from front to basal margins, through midline) 


PW
 width of pronotum (greatest width of pronotum) 


EL
 length of elytra (measured from base to apex of elytra, through suture) 


EW
 width of elytra (greatest transverse distance across both elytra) 


The depository abbreviations used in the text are as following:

CIB
 Collection of Dr. Ingo Brunk, Dresden, Germany 


CPM
 Collection of Mr. Pierre Morvan (Carentoir, France) 


MNHN
Muséum national d’Histoire naturelle, Paris, France 


SCAU
South China Agricultural University, Guangzhou, China 


## Taxonomy

### Characteristics of the *sarawakensis* species group

Large-sized and robust; body brown or yellowish brown, moderately convex; glabrous, impunctate on head and pronotum; head wide, eyes moderate large and prominent; mentum bisetose in most species (except for *Orthogonius sabahicus* sp. n. in which the mentum is asetose); apical margin of elytra broadly rounded, not truncate, and so without outer angles, inner angles broad in most species (but acute in *Orthogonius sarawakensis*); hind angles of pronotum widely rounded off, lateral expanded margins well-marked, subequal in width in middle, from flat (*Orthogonius sarawakensis*) to evidently reflexed throughout (*Orthogonius morvanianus* sp. n.); elytra convex, base well-bordered, striae moderately deep, intervals almost flat; 7^th^ interval simple, not carinated; at least median and posterior setiferous pores of the 3^rd^ interval located on median portion, instead of closing to the 2^nd^ stria as in most *Orthogonius* species, anterior pores present (but absent in *Orthogonius sabahicus* sp. n.); the 2^nd^–7^th^ intervals subequal wide in middle; protarsi more developed than meso- and metatarsi; middle tibiae not expanded in male; middle coxae asetose medially; apical spurs of hind tibiae long and sharp; hind femora moderately dilated, with two setae posteriorly; the 1^st^ and 3^rd^ hind tarsomeres longer than the 2^nd^ and 4^th^ respectively, the 4^th^ tarsomere deeply and asymmetrically emarginated, outer lobe longer than the inner; all tarsal claws pectinate; prosternal process well-bordered at apex (but unbordered in *Orthogonius sarawakensis*); male genitalia robust, notably expanded in median portion, deeply arcuate ventrally, and then gradually narrowed towards apex which is more or less pointed, or suddenly contracted at tip, dorsal opening long and large; the apical lamella long or short, broadly blunt at apex.

**Figure 1.**
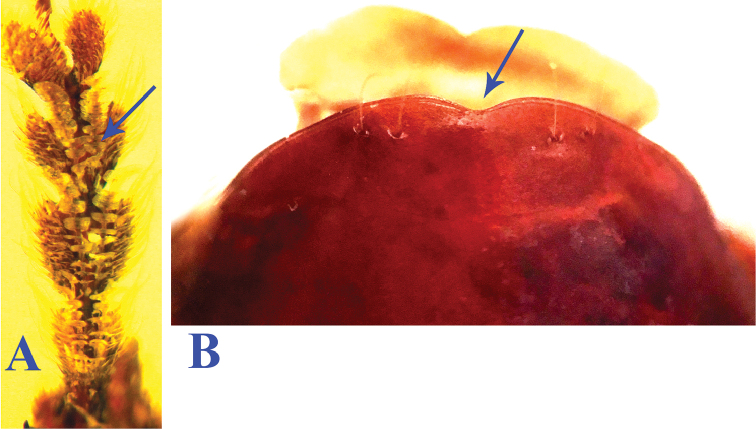
Protarsi and abdominal ventrite VII in male of *Orthogonius* **A** protarsi of *Orthogonius morvanianus* sp. n. **B** abdominal ventrite VII of *Orthogonius sarawakensis*.

**Sexual dimorphism.** In male, the 1^st^–3^rd^ protarsomeres with two rows of spongy setae ventrally (Fig. 1A); ventrite VII slightly and shallowly emarginated at apical margin (Fig. 1B).

**Distribution.** Southeast Asia.

### Key to species of the *sarawakensis* species group

**Table:** 

1	Median and posterior setiferous pores of the 3^rd^ elytral interval located on median portion, never close to the 2^nd^ elytra stria	**2**
–	Median and posterior setiferous pores of the 3^rd^ elytral interval close to the 2^nd^ elytra stria	**other *Orthogonius* groups**
2	Mentum asetose, the 3^rd^ elytral interval with only median and posterior setiferous pores, anterior one wanted	***Orthogonius sabahicus* sp. n.**
–	Mentum bisetose, the 3^rd^ elytral interval with three setiferous pores	**3**
3	Prosternal process unbordered at apex, inner angle of elytra sharp	***Orthogonius sarawakensis* Tian & Deuve, 2006**
–	Prosternal process well-bordered at apex, inner angle of elytra blunt	**4**
4	Labrum slightly emarginated at frontal margin, the 4^th^ hind tarsomere with a deeper emargination, outer lobe half as long as the joint	***Orthogonius perakicus* Tian & Deuve, 2007**
–	Labrum strongly emarginated at frontal margin, 4^th^ hind tarsomere with a shallower emargination, outer lobe shallower, about 1/3 as long as the joint	***Orthogonius morvanianus* sp. n.**

#### Orthogonius sarawakensis

Taxon classificationAnimaliaColeopteraCarabidae

Tian & Deuve, 2006

Figs 1B , 2A–C

Orthogonius sarawakensis Tian & Deuve, 2006: Tian and Deuve 2006: 133.

##### Type material.

**Figure 2.**
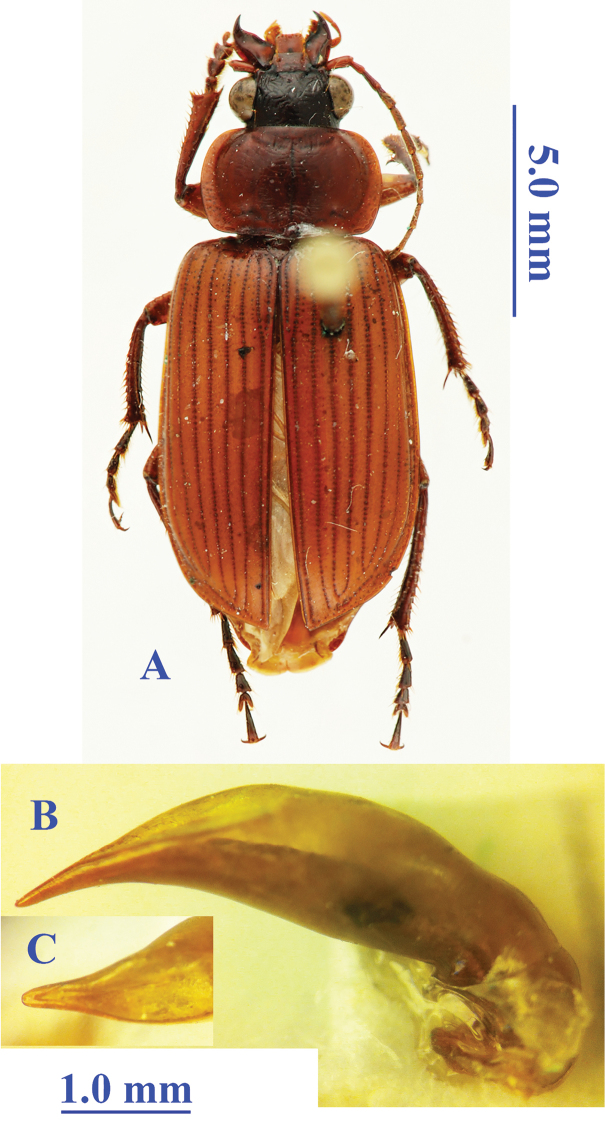
*Orthogonius sarawakensis* Tian & Deuve, 2006 **A** habitus, male, holotype **B** median lobe, right lateral view **C** apical lamella, dorsal view.

Length: 15.0 mm; width: 7.0 mm. Habitus as in Fig. 2A.

##### Diagnosis.

Head dark brown, other parts of the body yellowish brown; head stout, as long as wide; labrum slightly emarginated at frontal margin; clypeus even near base, with two longitudinal furrows along both side, joining to frontal impressions respectively; mentum bisetose; pronotum transverse, PW/PL = 1.55, widest at about middle, strongly convex; lateral expanded margins almost flat, equal wide throughout; elytra elongate ovate, EL/LW = 1.54; base well-bordered; the 3^rd^ interval with three setiferous pores, all are located on the interval; tarsal claws pectinate; prosternal process unbordered at apex, abdominal ventrite VII shallowly emarginated at apical margin in male.

Male genitalia (Fig. 2B, C): Short and robust, expanded in median portion; in dorsal view, apical lobe elongated, gradually narrowed towards apex, with apical lamella evidently longer than wide.

Female: Unknown.

##### Remarks.

Easily separated from its congeners, *Orthogonius perakicus* Tian & Deuve, 2007 and *Orthogonius morvanianus* sp. n., by its unbordered prosternal process at apex, pointed elytral apex, and more elongated apical lobe of aedeagus.

##### Material examined.

1 male, the holotype, “Nord Borneo, Mont Kina Balu, 5-8, 1903, John Waterstradt”, in MNHN; 1 male, same data as in holotype, in SCAU.

##### Distribution.

Malaysia (Sarawak).

#### Orthogonius perakicus

Taxon classificationAnimaliaColeopteraCarabidae

Tian & Deuve, 2007

Fig. 3

Orthogonius perakicus Tian & Deuve, 2007: Tian and Deuve 2007: 239.

##### Type material.

**Figure 3.**
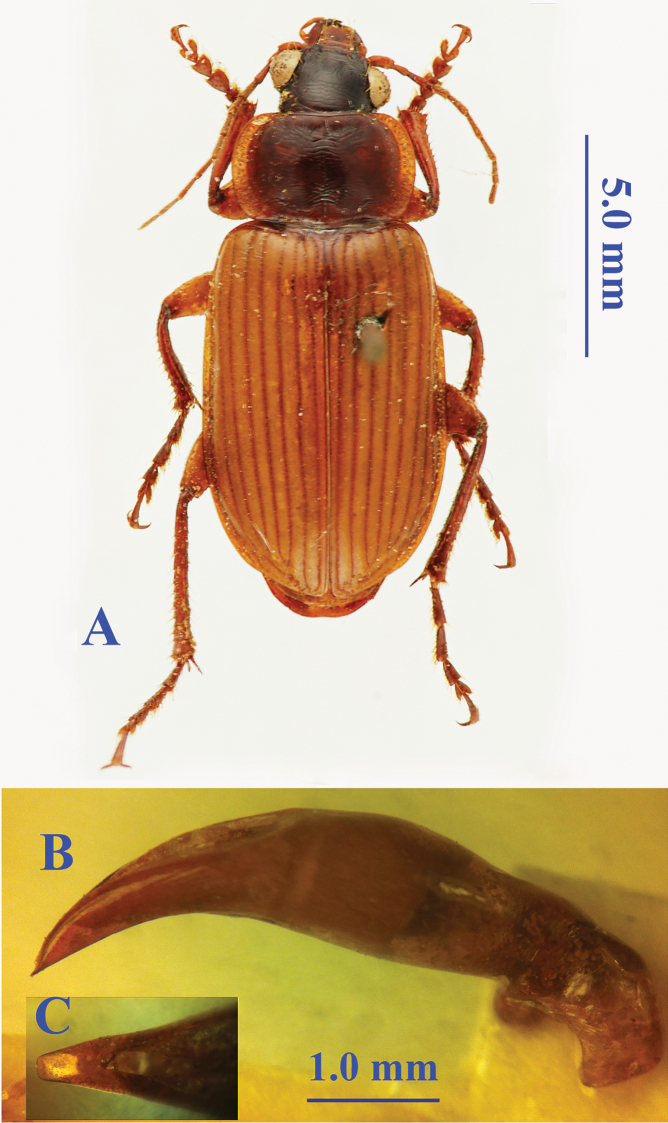
*Orthogonius perakicus* Tian & Deuve, 2007 **A** habitus, male, holotype **B** median lobe, right lateral view **C** apical lamella, dorsal view.

Length: 14.0 mm; width: 5.5 mm. Habitus as in Fig. 3A.

##### Diagnosis.

Elytra and sides of pronotum yellowish brown, head dark brown, pronotum light dark brown on disc; head as long as wide, clypeus smooth, front margin of labrum slightly emarginated; mentum bisetose; pronotum strongly transverse, PW/PL = 1.62; lateral expanded margins narrow and reflexed throughout, evenly widened; elytra well-bordered at base, elongate ovate, EL/EW = 1.58; apex broadly truncate, without outer angles, inner angles broadly obtuse; intervals flat; the 3^rd^ interval with three setiferous pores, all on median portion of the interval; legs moderately developed; the 4^th^ hind tarsomere emarginated as deep as 1/3 of the joint; all tarsal claws pectinate; prosternal process well-bordered at apex; abdominal ventrite VII in male slightly emarginated at apical margin.

Male genitalia (Fig. 3B, C): Median lobe of aedeagus stout, more or less bent near apex, apex pointed in profile, but obtuse in dorsal aspect, apical lamella longer than wide.

##### Remarks.

Allied to *Orthogonius morvanianus* sp. n. Differs from the latter by its labrum shallowly emarginated at front, the 4^th^ hind tarsomere having a deeper emargination, and apical lamella of aedeagus blunt at apex (see below for detail).

##### Material examined.

1 male, the holotype, “Perak”, in the Collection of Bates, MNHN.

##### Distribution.

Malaysia (Perak).

#### Orthogonius morvanianus sp. n.

Taxon classificationAnimaliaColeopteraCarabidae

http://zoobank.org/2695C6FD-230F-41D4-A765-25DFD75E49C2

Figs 1A , 4A–C

##### Holotype.

Male, “Thailand South, near Khao Sok NP, 3-6. II. 1997, A. Kudrna lgt”, in CPM.

**Figure 4.**
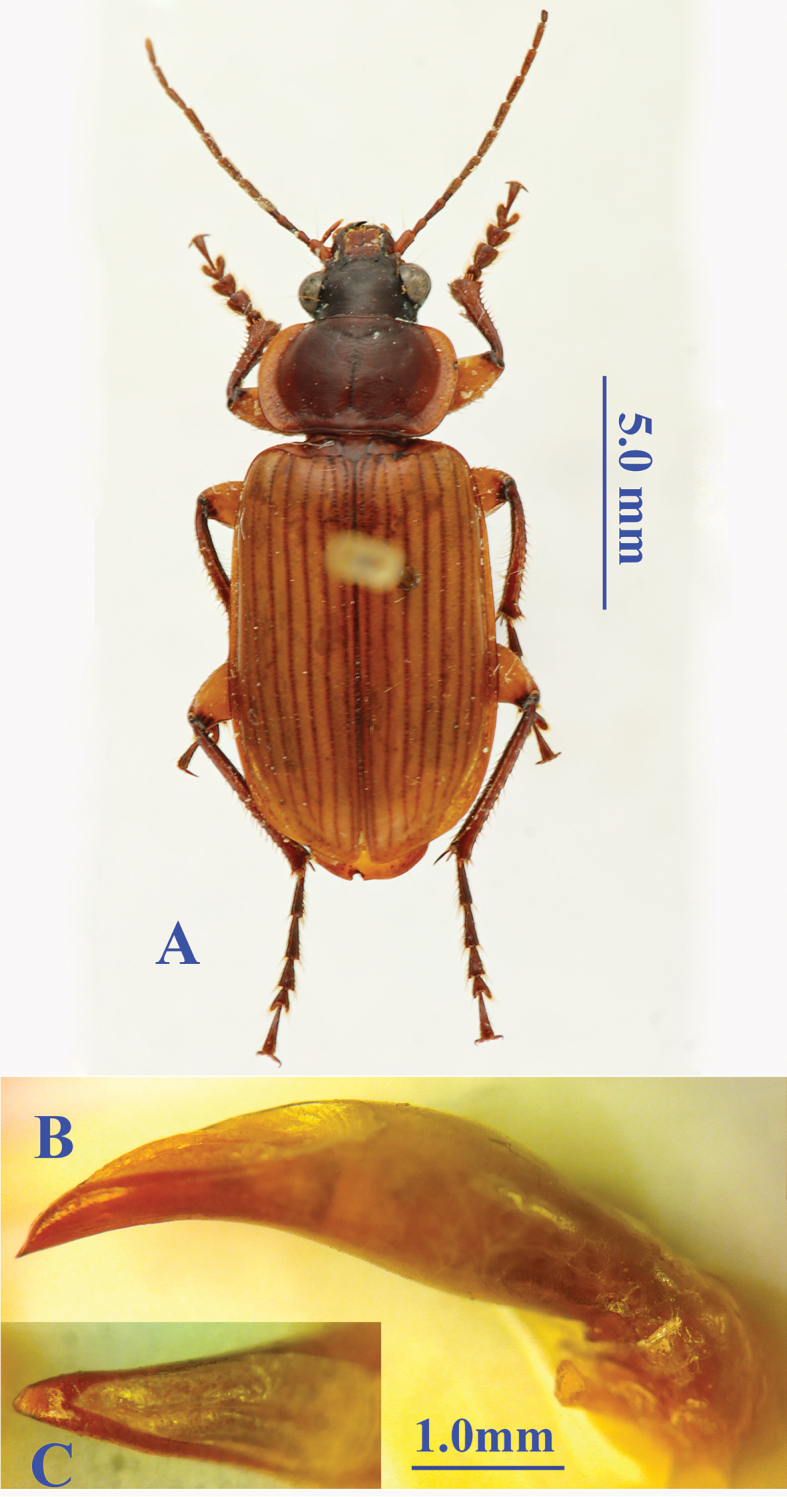
*Orthogonius morvanianus* sp. n. **A** habitus, male, holotype **B** median lobe, right lateral view **C** apical lamella, dorsal view.

Length: 14.0 mm; width: 5.5 mm. Habitus as in Fig. 4A.

##### Description.

Yellowish brown, with head dark brown, the 2^nd^–11^th^ antennomeres, disc of pronotum, tibiae and tarsi of legs brown; surface glabrous, impuctate, head and pronotum finely striate, the 3^rd^, 5^th^ and 7^th^ elytral intervals each with a few tiny sparse punctures; surface moderately shiny; microsculptural engraved meshes densely isodiametric on the surface of head, pronotum and elytra; body rather flat.

Head stout, as long as wide; eyes very large and prominent; frons flat, vertex slightly convex, smooth; frontal impressions very small, foveate; clypeus bisetose, rather smooth near base; labrum 6-setose, evidently emarginate at apical margin; ligula very small and narrow, bisetose at apex; palps slender or elongate, subcylindrical, normally setose; palpiger asetose, mentum edentate, mentum and submentum each with one pair of setae, mental ones much shorter; antennae slender and long, reaching 1/4 of elytra from base, pubescent from apical 2/3 of the 4^th^ antennomere; the 3^rd^ antennomere as long as the 4^th^, both shorter than the 1^st^; the 1^st^–3^rd^ antennomeres glabrous; evidently expanded laterally in the 4^th^ and 5^th^ antennomere; the 1^st^ antennomere 1.6 times as long as the 2^nd^.

Pronotum strongly transverse, PW/PL = 1.78, sides evenly rounded, widest at about middle; both basal and fore margins beaded; lateral expanded margins well defined, subequal wide throughout, strongly reflexed, smooth though with punctate-like structures; both fore and hind angles rounded off; disc moderately convex, fore transversal impression evident, while hind one well-marked, joining basal foveae and the lateral expanded margins.

Elytra elongate ovate, much longer than wide, EL/EW = 1.63, moderately convex, apex broadly truncate, without outer apical angle, inner apical angle broadly obtuse; basal borders well-bordered; sides more or less paralleled in middle portion; striae quite deep; intervals nearly flat, subequal in width; the 3^rd^ interval with three setiferous pores, anterior one close to the 3^rd^ stria, while other two, median and posterior ones, on median portion of the interval; the 7^th^ interval not carinated and without setae throughout.

Legs moderately developed; hind femur long and elongate, with 2 setae posteriorly; middle and hind coxae smooth and glabrous; fore tibia deeply sinuate at apical margin, outer angle sharp, outer margin distinctly serrate; middle tibia slender, not dilated medially in male; hind tibia slender, apex slightly dilated, apical spurs very long and sharp, the 1^st^ tarsomere slightly longer than the 2^nd^, the 3^rd^ tarsomere about 1.2 times longer than the 4^th^ which is bilobed at apex, with outer lobe slightly shorter than half as long as the joint, outer lobe evidently longer than the inner; all tarsal claws moderately pectinate.

Male genitalia (Fig. 4B, C): Robust, expanded in median portion, then deeply concave on ventral margin, apex suddenly contracted, sharp, dorsal opening long and large, apex more or less pointed, with a fine tooth at subapex; in dorsal view the apical lamella quite long, slightly longer than wide, roundly obtuse at apex.

Prosternal process bordered at apex; apical margin of abdominal ventrite VII feebly emarginated in male.

Female: Unknown.

##### Remarks.

Close to *Orthogonius perakicus* Tian & Deuve, 2007, but labrum strongly emarginated at frontal margin (slightly emarginated in *Orthogonius perakicus*), the 4^th^ hind tarsomere deeply emarginated, but with outer lobe about 1/3 as long as the joint (half as long as the joint in *Orthogonius perakicus*).

##### Etymology.

To be dedicated to Mr. Pierre Morvan (Carentoir, France), a good expert of Carabidae.

##### Distribution.

Southern Thailand.

#### Orthogonius sabahicus sp. n.

Taxon classificationAnimaliaColeopteraCarabidae

http://zoobank.org/C8EA2EDC-08D7-42E2-962E-347D379DCF29

Fig. 5

##### Holotype.

male, “Malaisie, Sabah, Crocker Range, Avril 94, Chew”, in MNHN; paratypes: 1 male, “Malaysia, N. Borneo, Sabah, Keningau distr. Trus Madi Mt. 1150 m, 7.VII.2011, A. Klimenko legit”, in CIB; 1 female, IBID, in SCAU.

**Figure 5.**
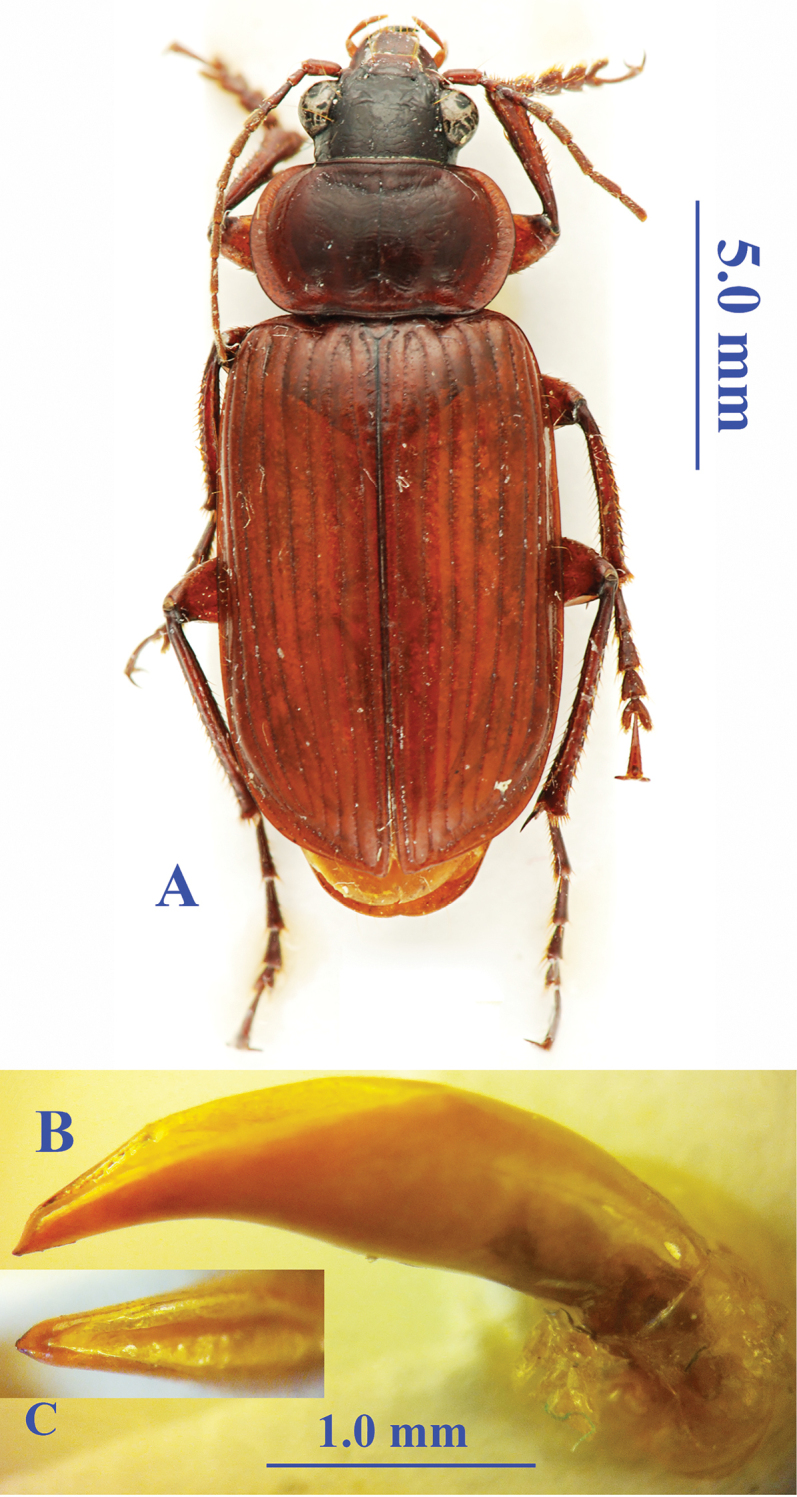
*Orthogonius sabahicus* sp. n. **A** habitus, male, holotype **B** median lobe, right lateral view **C** apical lamella, dorsal view.

Length: 16.0–17.0 mm; width: 6.5–6.6 mm. Habitus as in Fig. 5A.

##### Description.

Head dark brown, other parts reddish brown; smooth and glabrous, impunctate, elytral intervals covered with fine and sparse punctures; head and pronotum faintly wrinkled; microsculptural engraved meshes isodiametric on head, pronotum and elytra.

Head stout, slightly longer than wide, HL/HW = 1.05–1.10; vertex convex; labrum quite long, nearly as long as wide, widely and feebly emarginated at frontal margin, 6-setose; clypeus bisetose, base raised and sides longitudinal furrowed, joining to the frontal impressions; frontal impressions pit-like; eyes moderately developed and prominent; palps slender, subcylindrical, the 3^rd^ maxillary palpomere as long as the 4^th^, both glabrous, with two short setae at apex of the 3^rd^; the 2^nd^ labial palpomere slightly longer than the 3^rd^, bisetose on inner margin, the 3^rd^ faintly and sparsely setose; ligula narrow, bisetose at apex; mentum and palpigers unsetose, submentum bisetose; antennae extending beyond basal 1/5 of elytra, pubescent from the 4^th^ antennomere, starting from basal 1/4; the 1^st^–3^rd^ antennomeres and basal 1/4 of the 4^th^ antennomere glabrous; the 1^st^ antennomere stouter than other, with a long seta at subapex, several shorter setae present at apexes of each joint from the 2^nd^ antennomere; the 1^st^ antennomere less than twice as long as the 2^nd^, and slightly longer than the 3^rd^.

Pronotum transverse, notably wider than long, PW/PL = 1.66–1.68, widest at about middle; lateral expanded margins flat, even, feebly reflexed throughout; front and base well bordered; fore transversal impression faint, basal one evident; basal foveae small; disc markedly convex; front margin nearly straight at middle, basal margin feebly bisinuate; hind and front angles completely rounded off; base slightly shorter than front.

Elytra elongate-ovate, distinctly longer than wide, EL/EW = 1.61–1.63; nearly parallel-sided, widest at middle; basal border interrupted against the 1^st^–3^rd^ intervals; humeri rather square; apex roundly truncate, without outer angles, inner angle broadly blunt; disc convex; striae finely and deeply striate; intervals convex, subequal in width; the 3^rd^ interval with median and posterior setiferous pores (anterior one wanted), both are located on middle of the interval.

Femora moderately expanded, hind femur bisetose posteriorly; for tibia dilated at apex, apical margin obtusely truncate or sinuate towards outer angle, outer margin notably serrate; middle tibia slightly curved; hind legs slender, apical spurs long and sharp, the 1^st^ and 3^rd^ tarsomeres much longer than the 2^nd^ and 4^th^ respectively, the 4^th^ tarsomere deeply emarginated at apex, with lobes about half as long as the joint; fore and middle tarsal claws weakly pectinate, hind claws simple.

Prosternal process bordered at apex; abdominal ventrite VII finely and shallowly emarginate at apical margin in male.

Male genitalia (Fig. 5B, C): Stout and robust, apex suddenly narrowed and pointed in lateral view; the apical lamella short, wider than long.

##### Remarks.

It is a very peculiar species, differing from other congeners by its mentum asetose, elytral base incompletely bordered, anterior dorsal pores missed on the 3^rd^ interval, and hind tarsal claws simple.

##### Etymology.

To refer to the type locality.

##### Distribution.

Malaysia (Sabah).

## Supplementary Material

XML Treatment for Orthogonius sarawakensis

XML Treatment for Orthogonius perakicus

XML Treatment for Orthogonius morvanianus

XML Treatment for Orthogonius sabahicus

## References

[B1] Tian MY Deuve T (2006) Contribution to the knowledge of the tribe Orthogoniini of the Oriental Region (Coleoptera: Caraboidea) (I, II). Coléoptères 12(8, 9): 69–154.

[B2] Tian MY Deuve T (2007) Designations of the lectotypes for Bates’ species of the genus *Orthogonius* MacLeay (Coleoptera: Caraboidea: Orthogoniini), with descriptions of four new species from Bates’ Collection. Bulletin de l’Institut Royal des Sciences Naturelles de Belgique, Entomologie 77: 235–241.

[B3] Tian MY Deuve T (2013) Definition and review of the *lancangjiang* species group of the termitophilous genus *Orthogonius* MacLeay, 1825 (Coleoptera, Carabidae, Orthogoniini). ZooKeys 349: 81–100. doi: 10.3897/zookeys.349.6164 24294080 10.3897/zookeys.349.6164 PMC3837407

[B4] Tian MY Deuve T (2016) A review of the *baconii* species group of the termitophilous genus *Orthogonius* MacLeay (Coleoptera: Carabidae: Orthogoniini). Zootaxa 4093(1): 118–126. doi: 10.11646/zootaxa.4093.1.7 10.11646/zootaxa.4093.1.7 27394484

